# Engineering of band gap states of amorphous SiZnSnO semiconductor as a function of Si doping concentration

**DOI:** 10.1038/srep36504

**Published:** 2016-11-04

**Authors:** Jun Young Choi, Keun Heo, Kyung-Sang Cho, Sung Woo Hwang, Sangsig Kim, Sang Yeol Lee

**Affiliations:** 1 Korea University, Department of Electrical Engineering, Seoul, 136-701, Republic of Korea; 2 Samsung Advanced Institute of Technology, Device Lab, Suwon-si, 443-803, Republic of Korea; 3 Cheongju University, Department of Semiconductor Engineering, Cheongju, 360-764, Republic of Korea

## Abstract

We investigated the band gap of SiZnSnO (SZTO) with different Si contents. Band gap engineering of SZTO is explained by the evolution of the electronic structure, such as changes in the band edge states and band gap. Using ultraviolet photoelectron spectroscopy (UPS), it was verified that Si atoms can modify the band gap of SZTO thin films. Carrier generation originating from oxygen vacancies can modify the band-gap states of oxide films with the addition of Si. Since it is not easy to directly derive changes in the band gap states of amorphous oxide semiconductors, no reports of the relationship between the Fermi energy level of oxide semiconductor and the device stability of oxide thin film transistors (TFTs) have been presented. The addition of Si can reduce the total density of trap states and change the band-gap properties. When 0.5 wt% Si was used to fabricate SZTO TFTs, they showed superior stability under negative bias temperature stress. We derived the band gap and Fermi energy level directly using data from UPS, Kelvin probe, and high-resolution electron energy loss spectroscopy analyses.

Amorphous oxide semiconductors (AOSs) have gained much attention over the past decades as candidate materials for next-generation thin film transistors (TFTs). These materials have applications in integrated multilayered microelectronics, flat panel displays, and flexible displays^1^,^2^,^3^. Currently, great efforts have been made to enhance the performance and the stability of oxide TFTs by optimising the gate insulator, electrode, active channel layers, and TFT structure^4^,^5^,^6^,^7^,^8^. In particular, amorphous InGaZnO (a-IGZO) has been extensively investigated due to its favourable properties, including high field effect mobility, large area uniformity, and long-term stability^9^,^10^,^11^. Numerous studies have reported AOS TFTs with several multicomponent oxide semiconductors, such as InGaZnO, ZnSnO, and HfGaZnO^12^,^13^,^14^. Recently, indium free materials, like zinc-tin oxide (ZTO), have been extensively studied for the use of active channel layer of TFTs. Oxide-based multicomponent semiconductors have several advantages over conventional Si-based semiconductors, such as visible light transparency, large area deposition at low temperature, and high carrier mobility. For ZTO TFTs, the high mobility comes from an increase in the population of charge carriers (electrons) originating from oxygen vacancies. However, too many deep trap levels within the band gap can lead to instability of the device during operation^15^,^16^, such as large off currents and a depletion mode with a large threshold voltage shift^17^,^18^,^19^.

For high performance and stable device operation, it is important to optimize the O-deficiency and deep state formation^20^,^21^,^22^,^23^. In a previous study, it was found that the Si atoms could suppress the oxygen deficiency, due to the high bonding strength with oxygen^17^. Therefore, doping with Si atoms can be a strategy to enhance the electrical characteristics as well as the device stability. However, the role of the Si dopant atoms in the ZTO films regarding TFT device performance and stability is not yet clear. Investigation of the basic semiconducting properties, such as the Fermi-level and energy band gap configuration, including the position of the valence band maximum (VBM) and the conduction band minimum (CBM) are key properties related to the carrier concentration and the mobility of the films for AOS TFT operation. In this study, we examined Si doped ZTO TFTs in which the active semiconductor layers were AOS thin films and the Fermi energy level could be controlled by changing the Si doping ratio. To investigate the effect of Si doping on the ZTO semiconductor, the energy band diagrams were carefully derived by combining the results of Kelvin probe microscopy (KP), ultraviolet photoelectron spectroscopy (UPS), and high-resolution electron energy loss spectroscopy (HR-EELS) measurements. In addition, to investigate the effect of Si doping of ZTO on the electrical characteristics of SZTO TFTs, a series of TFTs with different Si ratios were fabricated and characterized in terms of the Si doping concentration.

## Results and Discussion

**Figure 1:**
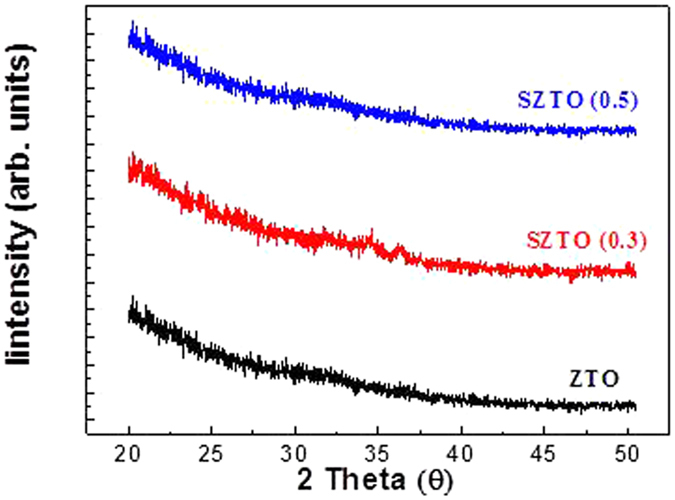
XRD pattern of the SZTO films deposited on silicon substrate, with different Si concentration.

**Figure 2:**
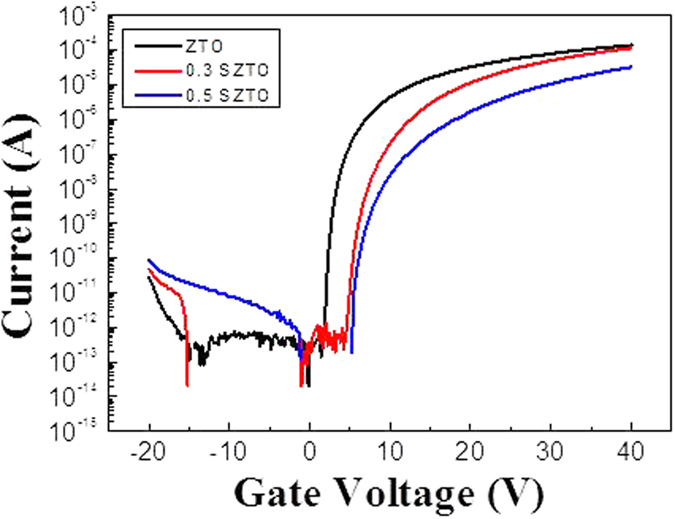
Transfer characteristics at VDS = 5.1 V for the SZTO TFT with different Si contents. The TFT is formed on a 200-nm-thick a-SiO2/n+-Si wafer with a top-contact structure.

**Figure 3:**
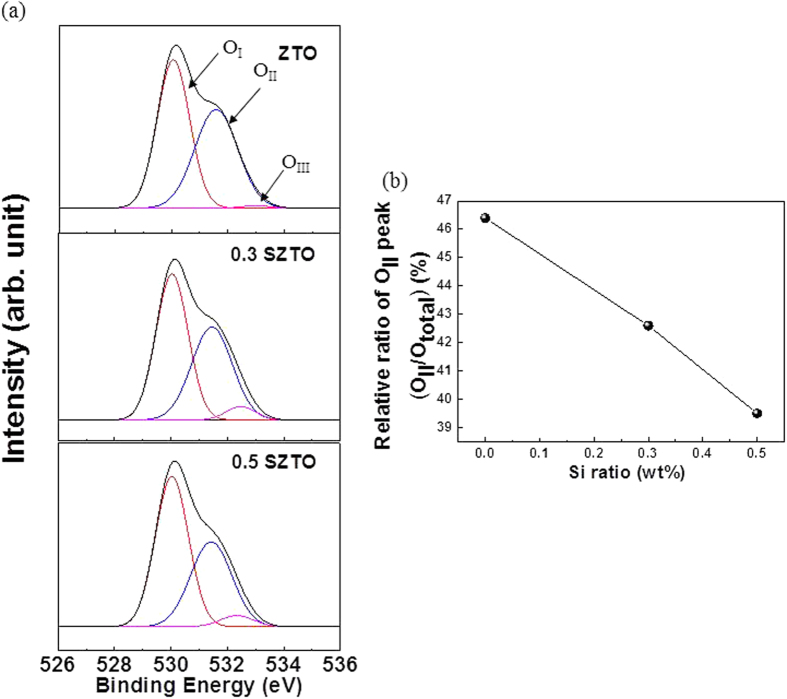
XPS spectra of the O1s core level line for the, (**a**) ZTO, 0.3 SZTO and 0.5 SZTO films and relative ratio of OII peak (OII/Ototal) as a function of Si ratio. (**b**) The relative concentration of OII peak in SZTO films as a function of Si ratio. All calculations are performed based on an area integration of each O1s peak.

**Figure 4:**
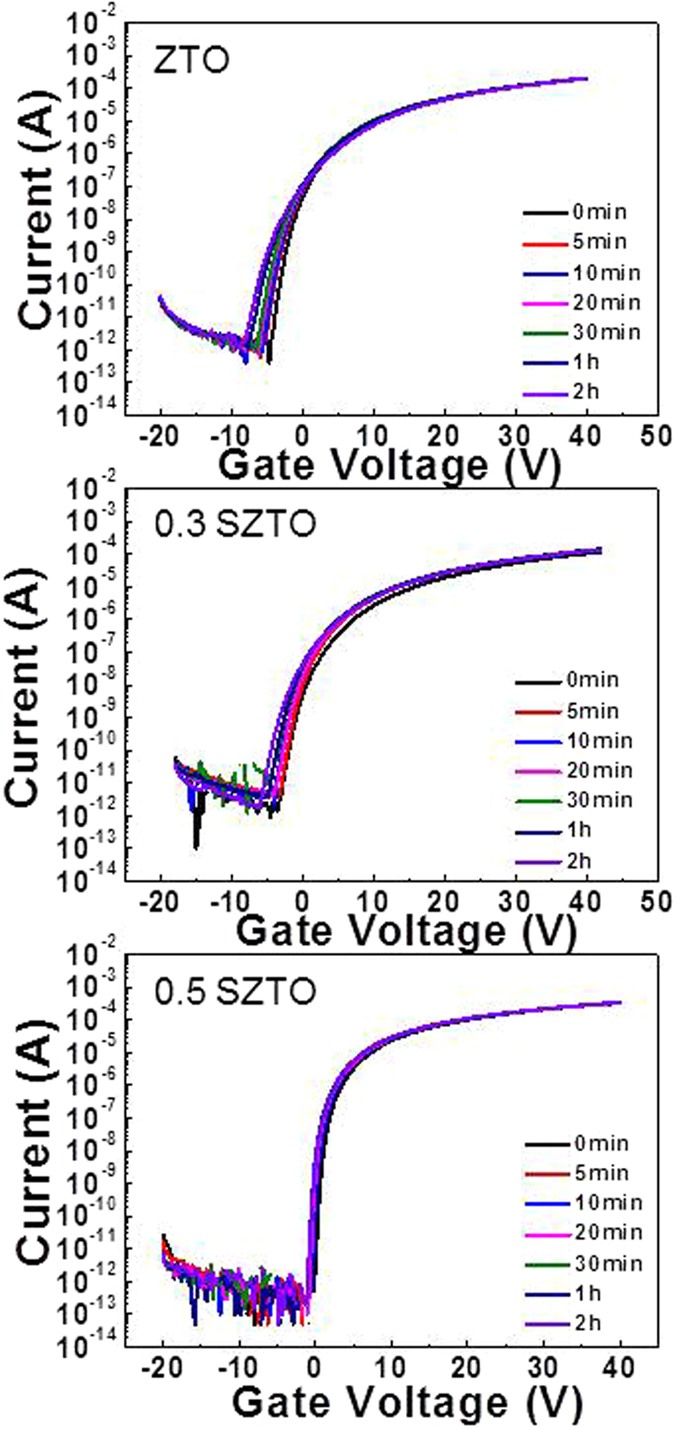
Variations in the transfer characteristics of ZTO and SZTO TFTs under the NBTS test. VGS = −20 V and VDS = 10.1 V, and T = 60oC, for a duration of 2 h.

**Figure 5:**
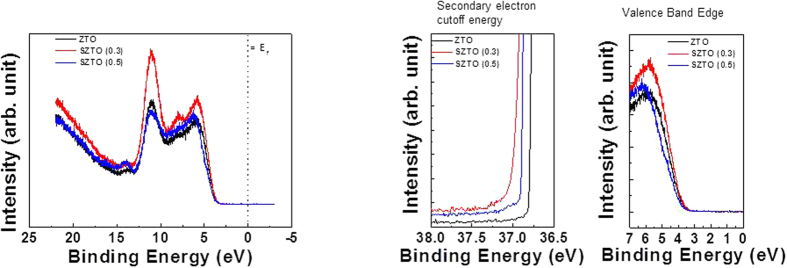
(**a**) Experimental UPS spectrum of the ZTO and SZTO (0.3 and 0.5 wt %) (**b**) He II spectra of secondary electron cutoff and valence band edge with varying Si contents.

**Figure 6:**
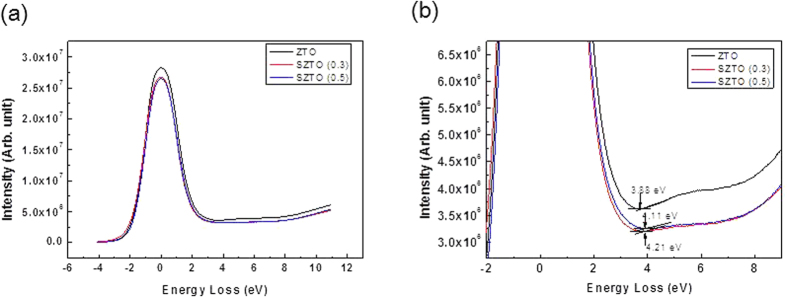
EELS spectra of a SZTO showing (**a**) the plasmon loss peaks and (**b**) the band-gap measurements at various Si contents. Note that as the silicon contents increases, the band-gap also increases.

**Figure 7:**
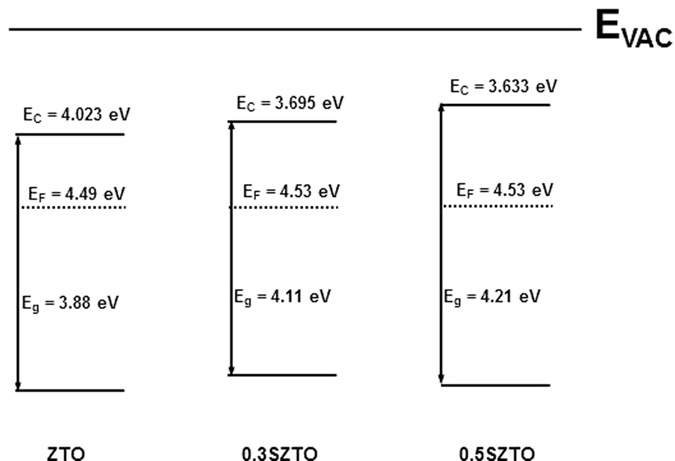
Calculated band-alignment energy diagrams indicating the relative energy position of conduction band, valence band, and Fermi level for Si-doped ZTO with various Si contents. The vacuum level is set to 0 eV in each case.

The diffraction patterns of the SZTO layers revealed typical amorphous behaviour, as shown in Fig. 1; A (undoped ZTO), B (0.3 wt% SZTO), and C (0.5 wt% SZTO). The ZTO and SZTO thin films directly deposited on the SiO_2_ layer on the substrate showed an amorphous phase (no peaks were observed). In addition, the addition of Si atoms increased the average crystallite size of SZTO films, resulting in an increased surface roughness^24^. The root-mean-square surface roughness of films A, B, and C were 0.38, 0.46, and 0.48 nm, respectively. Figure 2 shows the electrical properties of undoped and doped SZTO TFTs with different amounts of Si from current-voltage (I-V) curves. The field effect mobility of samples A, B, and C decreased from 14.9 cm^2^/V s to 7.9 cm^2^/V s, respectively. Table 1 summarizes the electrical characteristics of Si doped ZTO TFTs depending on various Si ratio. The dependence of the electrical properties on the silicon ratio could be related to the defect concentration in the thin films. Oxygen vacancies, acting as trap states in the oxide materials, deteriorate the electrical properties, leading to a large shift in the threshold voltage, low mobility, and high sub-threshold swing^25^. Typically, the addition of Si atoms decreases the carrier concentration of an oxide semiconductor due to the high bond strength of Si-O (799.6 kJ/mol) compared with those of Sn-O (531.8 kJ/mol) and Zn-O (395 kJ/mol)^10^. In previous work, we investigated the electronic structure of the present SZTO compound and the energetic formation of the oxygen deficient state through first principles calculations^26^. The effective mass of an electron carrier (m*) tends to increase with increasing Si concentration, meaning that the electronic structure sensitively responds to any lattice fluctuation related with a change in the conduction band position. To understand the origin of the change in the electrical properties, the chemical states of the films were examined by X-ray photoemission spectroscopy (XPS). Figure 3(a) shows the O1s XPS spectra for films A, B, and C. The binding energy of photoelectrons was calibrated using the C1s peak at 284.25 eV as a reference. The O1s peak on the surface was fitted by three Gaussians peaks; labelled (O_I_), (O_II_), and (O_III_). The lowest energy sub-peak (O_I_) at 530.25 eV was assigned to the oxygen-binding region of the matrix without oxygen vacancies, and the medium energy sub-peak (O_II_) at 531.62 eV is associated with the oxygen-deficient regions^27^,^28^. The highest energy sub-peak (O_III_) at 532.5 eV is related to the metal hydroxide peak^29^. The density of the oxygen-deficient bonding state (O_II_) decreased with increasing Si ratio, as shown by the relative ratios of the O_II_ peak in Fig. 3(b). The relative ratio of the O_II_ peaks of films A, B, and C decreased from 46.4% to 39.5%, respectively. This result clearly indicates that the concentration of oxygen vacancies (V_O_) decreased, probably resulting in a decrease in the carrier concentration with increasing Si content. Even though the individual contribution of V_O_ to the electrical performance was not quantified precisely, the formation of V_O_ results in the generation of charge carriers due to the increase of free electrons, explained by the electron charge trapping model. Figure 4 shows the evolution of the transfer curves over time obtained at a drain-to-source voltage (V_DS_) of 5 V for films A, B, and C under negative bias temperature stress (NBTS). The NBTS was kept at a gate-to-source voltage (V_GS_) of −20 V and V_DS_ of 10.1 V over 7,200 s at 60 °C. The formation of O-deficient states is easily suppressed by Si-doping due to the strong binding energy with oxygen^30^,^31^. SZTO TFT (0.5 wt%) showed a threshold voltage shift (ΔV_th_) of 0.9 V. The strong bonding strength of silicon suppressed V_O_ formation, resulting in a decrease in the carrier concentration. However, there are problems explaining the behaviour of the electrical characteristics of oxide semiconductors using only the O1s peak mechanism. The O1s peak analysed by XPS can only explain the oxygen related defects in the metal-oxide structure, such as the occupied electron trap states in the oxide semiconductor. It has been proposed that the oxygen deficiency generated by the removal of oxygen atoms separated from the lattice primarily produces deep and localized states near the VBM^32^. Moreover, Duke *et al*.^32^,^33^,^34^,^35^ suggested a reconstructed model of the prism face. In addition, it could be expected that we could control the electrical performances and the stability of TFTs by changing the band offset. This implies that the addition of Si atoms can control the Fermi level related with the change in carrier concentration in amorphous oxide n-type semiconductors. Therefore, it is very important to derive the energy band diagram to investigate any changes in the electron carrier concentration and explain the behaviour of the electrical characteristics. To obtain further insight into the band gap structure of SZTO TFTs with different Si ratios, a typical valence band was measured by UPS. He (II) UPS spectra are shown in Fig. 5. The secondary electron cut-off energy (SC) and the valence band edge energy (E_VBE_) were derived using a linear extrapolation of the leading edges to the backgrounds of the spectra. From these extracted values, the valence band energies were obtained analogously using equation (1)^36^,


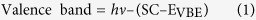


where *hv* is the incident He (II) line energy at 40.813 eV^37^ and E_VBE_ is defined as the energy separation between the VBM edge and the Fermi level. It is clear that the valence band spectra changed with Si doping of the SZTO system. In order to elucidate the origin of the observed band-gap changes, HR-EELS spectra were obtained using a 1.5 keV exciting electron beam, as shown in Fig 6(a). HR-EELS is capable of analysing electronic and optical properties of oxide materials because the low-energy-loss region reflects the valence and conduction band structures of solids. All spectra showed a strong elastic peak, followed by a flat and featureless region and then a broad energy loss peak. The onset of the energy loss peak in the spectrum gives the energy gap corresponding to that particular scattering geometry. The band-gaps of films A, B, and C were estimated by the linear fit method (Fig. 6(b)), yielding values of 3.88, 4.11, and 4.21 eV, respectively. As the Si ratio increased, the bandgap widened. We also used KP to measure the work function of SZTO thin films, where the measured contact potential difference was calibrated using an *in situ* sputtered Au reference sample^38^,^39^. The valence band and band gap values calculated from UPS and HR-EELS allowed us to estimate the position of the conduction band (E_c_)^40^. The experimentally determined band gaps are shown in Fig. 7. Thecorresponding values of the band gaps of ZTO and SZTO (0.3, 0.5 wt %) thin films were 3.88, 3.82, and 4.09 eV, respectively. The band gap and energy level values are reported in Table 2 for each oxidethin film. The field effect mobility decreased from 14.9 to 7.9 cm^2^/V s with doping mainly caused by the decrease of the electron carrier concentration at 0.3 and 0.5 wt% Si content. It has been reported that a low carrier concentration can lead to low mobility in oxide TFTs due to the suppression and passivation of oxygen vacancies^41^. However, it is still uncertain why the addition of Si changed the electrical characteristics. After adding Si atoms (0.3 and 0.5 wt %), a drastic increase in the ΔE_CB_ from 0.467 to 0.897 eV was observed^42^. The wave function of the CBM is characterized mainly by Sn-5s and Zn-4s orbitals in the ZTO thin film. That is, increasing the CBM relative to the vacuum level will lead to an increase in the work function, provided that the Fermi level stays close to the CBM. Actually, the Si^4+^ ions incorporated in the ZTO matrix do not generate carriers in the SZTO, because the Si^4+^ ions substituted into the Sn^4+^ ion sites may work as oxygen binders for oxygen out-diffusion, thereby suppressing the formation of oxygen vacancies. This oxygen-related defect model has usually been explained as the electron charge trapping mechanism in oxide TFTs^43^,^44^. The carrier transport is governed by percolation conduction over trap states and is enhanced at high carrier concentrations by filling the trap states. The decrease in the carrier concentration is explained by the increase in ΔE_CB_. Therefore, the role of Si ions as oxygen binders in the SZTO materials is the reason for the stability improvement and band edge changes in the SZTO TFT.

In conclusion, the control of the carrier concentration and mobility in SZTO TFTs has been demonstrated, where the incorporation of Si suppresses the carrier concentration. However, a small amount of Si doping in SZTO TFTs improves the electrical properties due to a decrease in ΔE_CB._ We observed that the Si atoms in SZTO TFTs significantly affected the electrical properties. This investigation of the band gap characteristics of SZTO TFTs through UPS, KP, and EELS analyses provided a sufficient guideline for determining favourable oxide TFT characteristics leading to improved electrical properties.

## Conclusion

In summary, the relationship between the change of the energy band gap due to doping suppressors and the stability of TFTs due to oxygen vacancy has been verified. We have derived energy band diagrams for ZTO and SZTO thin films using EELS, UPS, and KP measurements. The effect of Si on the device performance of ZTO and SZTO TFTs has been systematically investigated. Increasing the Si content resulted in a change in the electrical conduction properties due to changes in the band structure. The work function of ZTO TFTs was found to be strongly dependent on the Si content. As the Si concentration increased, the Fermi level of the SZTO TFTs decreased. In addition, widening of the ΔE_CB_ relative to the vacuum level resulted in an increase in the work function. Si doping affected the band alignments including the band gap and CBM, VB, and E_f_, which in turn changed the electrical characteristics. Direct derivation of the energy band diagram could explain the enhancement of the stability of the TFTs due to the suppression of oxygen vacancies, resulting in a decrease in the defects in the band gap and carrier generation.

## Methods

### Device fabrication

First, a typical ultrasonic wafer cleaning process was used. SZTO films were deposited on Si wafers with a 200 nm thickness of SiO_2_ by radio frequency (RF) magnetron sputtering at room temperature followed by a standard lithography and wet etching process to pattern the film. Ceramic targets of SZTO (diameter of 2 in) were prepared by sintering mixtures of Si, Zn and Sn powders. The Si contents of the sputtering targets were 0, 0.3, and 0.5 wt.%, where the Zn: Sn ratio was fixed at 1:1. SZTO films of 50 nm thickness were deposited by RF magnetron sputtering at a processing pressure of 0.6 Pa and a sputtering power of 50 W. Photolithography and wet-etching was then performed and the width/length (W/L) of the active layer was patterned with 250/50 μm. After developing the photoresist, the source and drain electrodes were deposited using Ti/Au (10/50 nm) using an e-beam or thermal evaporation process, respectively. Annealing of the fabricated devices was carried out in N_2_ at 500 °C for 2 h. A poly methyl-methacrylate (PMMA) layer was deposited as a passivation layer over the channel layer by spin coating.

### Characterisation

All current-voltage (I-V) measurements were conducted using a semiconductor parameter analyser (EL423, ELECS Co.) at room temperature in a dark box. The crystallinity of the SZTO films was analysed using X-ray diffraction (XRD, modified Philips-1880) using CuKα radiation. The film morphology was observed using a JEOL JSPM-5200 atomic force microscope (AFM). UPS measurements were conducted in ultra-high vacuum (~10^−10^ mbar) where the samples were irradiated with 40.813 eV photons (He II line). For each sample, the work function was calculated from the UPS spectrum by subtracting the energy of the incident beam from the difference between the Fermi edge and the low-energy cut-off of secondary electrons. A commercial Kelvin probe system was used to measure the work function changes of the SZTO films. This apparatus measures the contact potential difference between a reference plate and the surface of silicon indium zinc oxide (SIZO). The 10 mm diameter probe plate was made of stainless steel and was electrically connected via ground to the sample during the measurement.

## Additional Information

**How to cite this article**: J. Y. Choi *et al*. Engineering of band gap states of amorphous SiZnSnO semiconductor as a function of Si doping concentration. *Sci. Rep.* **6**, 36504; doi: 10.1038/srep36504 (2016).

**Publisher’s note:** Springer Nature remains neutral with regard to jurisdictional claims in published maps and institutional affiliations.

## Figures and Tables

**Table 1:** Electrical parameters of SZTO transistors with different Si compositions.

Si: Zn: Sn	V_th_ (V)	I_on/off_ current ratio	μ_FE_ (cm^2^ V^−1^ S^−1^)	Sub-threshold swing (V/decade)
ZTO	3.5	2.5 × 10^8^	14.99	0.59
0.3 SZTO	1.5	2.4 × 10^7^	17.043	0.63
0.5 SZTO	6.7	9.2 × 10^6^	7.953	0.96

**Table 2:** Band gap parameters of ZTO thin films with different Si compositions.

	Bandgap	E_F_ (eV)	VB (eV)	CB (eV)	CB-EF
ZTO	3.88	4.49	7.903	4.023	0.467
0.3 SZTO	4.11	4.53	7.805	3.695	0.835
0.5 SZTO	4.21	4.53	7.843	3.633	0.897
